# Supplementary material for: Distributions of Invasive Arthropods across Heterogeneous Urban Landscapes in Southern California: Aridity as a Key Component of Ecological Resistance
Source: Insects. 2019 Jan 15;10(1):29. doi: 10.3390/insects10010029 (PMC6358729; doi:10.3390/insects10010029)
Supplement: Supplementary file 1 [file insects-10-00029-s001.pdf]

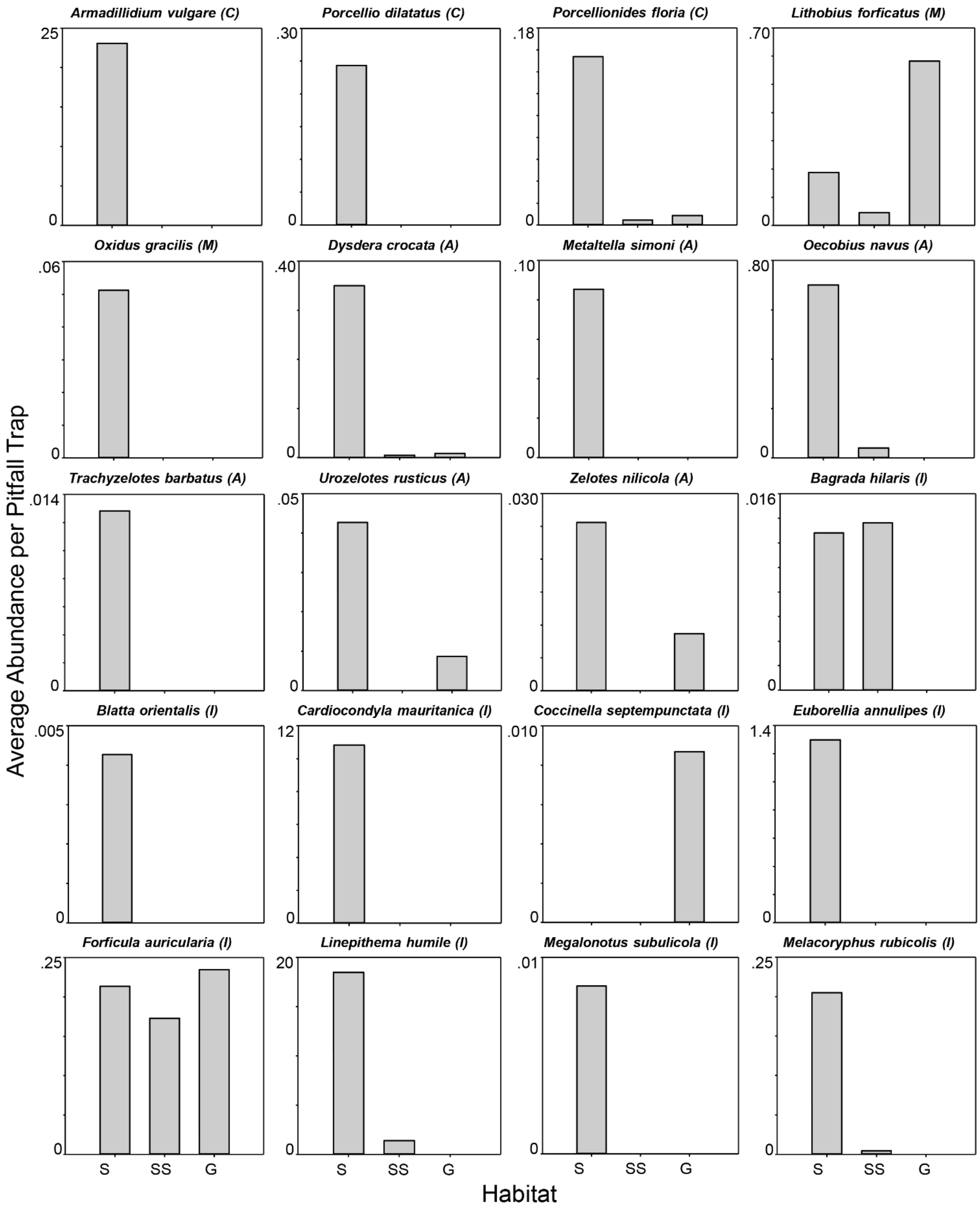

Supplemental Figure 1. Abundance of each non-native species. Bar height is the mean number of individuals per pitfall trap in each habitat type (S = Suburban; SS = Sage scrub; G = Grassland) over the five sampling periods. Letters next to species names indicate subphylum or class: C = Crustacea; M = Myriapoda; A = Arachnida; I = Insecta.
